# ﻿A new species of *Pseudechinopsyllus* George, 2006 (Copepoda, Harpacticoida, Cletodidae) from Campos Basin, Brazil (southwest Atlantic Ocean), with remarks on the phylogeny of the genus

**DOI:** 10.3897/zookeys.1241.153012

**Published:** 2025-06-19

**Authors:** Kai Horst George, Adriane P. Wandeness, Paulo J. P. Santos

**Affiliations:** 1 Senckenberg am Meer Wilhelmshaven, Abt. DZMB, Südstrand 44, D-26382 Wilhelmshaven, Germany Senckenberg am Meer Wilhelmshaven Wilhelmshaven Germany; 2 Departamento de Zoologia, Universidade Federal de Pernambuco, Recife, PE, Brazil Universidade Federal de Pernambuco Recife Brazil

**Keywords:** Cletodinae, Crustacea, deep sea, meiofauna, morphology, systematics, taxonomy

## Abstract

The discovery and description of a new representative of the *Ceratonotus* group (Copepoda, Harpacticoida, Cletodidae T. Scott) in the Grussaí Canyon off the coast of Brazil opened up the possibility of clarifying the relationship between *Echinopsyllus* Sars and *Pseudechinopsyllus* George. *Pseudechinopsyllusandrei***sp. nov.** is characterised by several autapomorphies, including (a) the extensive development of long spinules on the second and third segment of the female antennule, and the strong respectively extreme elongation of (b) the exopod and (c) the setophore on the female P5. Synapomorphies shared with *P.sindemarkae* include (d) the formation of long spinules at the bases of P2–P4 as well as (e) on exp1 of the P2, and (f) the strong elongation of a tube pore on P2 exp2. Furthermore, the sister group relationship of *Pseudechinopsyllus* and *Echinopsyllus* could be substantiated by eleven synapomorphies, among them (g) the large body size, (h) the development of long, rigid cephalothoracic ventrolateral anterior and posterior processes and (i) the loss of the syncoxal seta on the maxilliped. The present study represents a further step towards clarifying the systematic relationships within the *Ceratonotus* group. A key to the species is provided.

## ﻿Introduction

The present contribution deals with material collected during the research project “Campos Basin Environmental Heterogeneity – HABITATS” sponsored by the Brazilian petroleum company PETROBRAS (cf. George et al. 2013). Samples of meiofauna from the Campos Basin (Brazil) revealed many new species, providing important results that increase our knowledge of the biodiversity of meiofauna along the Brazilian coast, including animals of the taxon Cletodinae T. Scott, 1905 (cf. Wandeness et al. 2009; George et al. 2013).

This study adds and describes a new species of the formerly monotypic genus *Pseudechinopsyllus* George, 2006 (Copepoda, Harpacticoida, Cletodidae T. Scott, 1905). With *P.sindemarkae* George, 2006 the genus was first reported from the Great Meteor Seamount in the subtropical north-east Atlantic Ocean (George 2006a). *Pseudechinopsyllus* is assigned to the *Ceratonotus* group (George 2006a, 2020, 2021), which was transferred by George (2020) from the family Ancorabolidae Sars, 1909 to the Cletodidae and there to the Cletodinae. The *Ceratonotus* group currently comprises 13 different genera, seven of which, including *Pseudechinopsyllus*, are so far monotypic, i.e., represented by only one species (cf. George 2020, 2021). In the Grussaí Canyon (Campos Basin, Brazil), however, 11 individuals found proved to be a new species of that genus. They share many of the features of *P.sindemarkae* mentioned by George (2006a) but also have a number of features that characterise them as members of a distinct species that is described here and named *Pseudechinopsyllusandrei* sp. nov. Its discovery and placement in *Pseudechinopsyllus* enabled the phylogenetic characterisation of that genus and its justification as a monophylum. For this purpose, a morphologically based phylogenetic analysis was conducted based on 41 characters. Most selected characters were recognised as exclusive and unambiguous synapomorphies of the two *Pseudechinopsyllus* species on the one hand and the two monophyla *Pseudechinopsyllus* and *Echinopsyllus* on the other, compared with the remaining representatives of the *Ceratonotus* group. The results of the phylogenetic analysis are presented and discussed here in detail. Derived attributes that also occur as potential synapomorphies or convergences in particular taxa of the *Ceratonotus* group were not considered, except for six characters. These were incorporated because they are of particular importance for the characterisation of the taxa studied here. An ongoing comprehensive phylogenetic analysis of the entire *Ceratonotus* group including all potential convergences will be published elsewhere.

The discovery of *Pseudechinopsyllusandrei* sp. nov. extends the geographical range of *Pseudechinopsyllus* from the northern subtropical to the tropical southwest Atlantic Ocean. The bathymetric distribution of the genus has also expanded from ca 400 m (*P.sindemarkae*) to 1,300 m (*P.andrei* sp. nov.).

## ﻿Materials and methods

**Figure 1.**
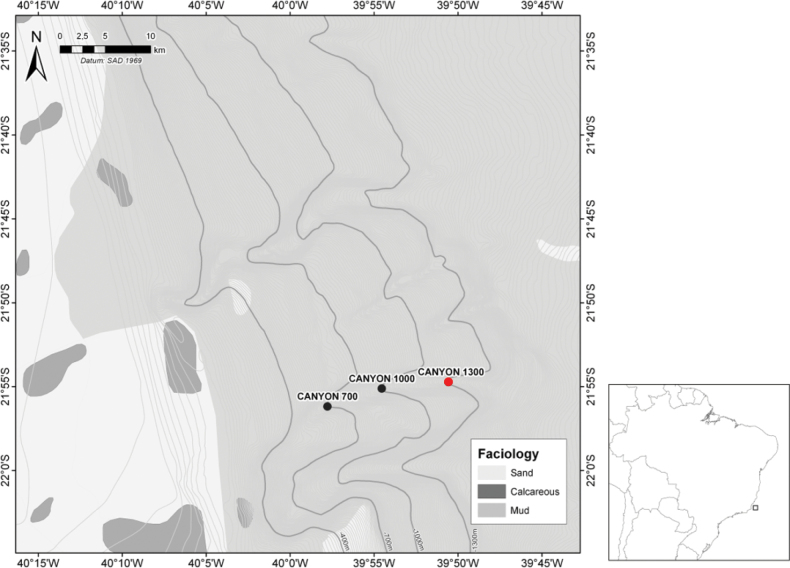
Map showing the Grussaí Canyon (Campos Basin, Brazil) and the stations at 700 and 1,000 m (black circles) as well as the type locality of *Pseudechinopsyllusandrei* sp. nov. (1,300 m water depth; red circle), sampled during the HABITATS project (2008, 2009).

Sampling was conducted during cruises of RV GYRE and RV EMMA MCCALL (HABITATS project, (2008, 2009). A total of eight stations along the 700, 1,000 and 1,300-m isobaths were sampled at the Grussaí Canyon, Campos Basin, Brazil, south-western Atlantic (Fig. 1). On board, samples were taken with a box corer containing 0.25 m^2^ of sediment subdivided into 25 subsamples. Each sample was transferred to a 1-l plastic flask and fixed in 10% formalin buffered with borax. Meiobenthic organisms were extracted by colloidal silica flotation (Giere 2009).

The harpacticoid copepods found in the samples were picked out and preserved in ethanol for later identification to species level. Dissected specimens were mounted on several slides with glycerine. The preparations were sealed with transparent nail varnish. All drawings were made using a camera lucida on a Leica DMR microscope equipped with differential interference contrast. They were then digitally traced using ADOBE Illustrator CS6.

The type material of *Pseudechinopsyllusandrei* sp. nov. comprises 11 specimens (7 females, 3 males, 1 copepodid CIV). All specimens are more or less severely damaged, which made it impossible to describe the species on the basis of a single individual. Consequently, we have refrained from naming a holotype and paratypes. Instead, all individuals are labelled as syntypes.

For Confocal Laser Scanning Microscopy (CLSM) syntype 2 (female) was used. As described by George et al. (2020), the specimen was stained overnight with a 1:1 solution composed of Congo Red and Acid Fuchsin adapted from Michels and Büntzow (2010). Subsequently it was mounted in a drop of glycerine placed in transparent, self-adhesive reinforcement rings to prevent direct contact between the specimen and coverslip. The images were generated using a Leica TCS SP5 consisting of a Leica DM5000 B upright microscope and three visible-light lasers. The used software was LAS AF 2.2.1 (Leica Application Suite Advanced Fluorescence). The images were taken with an objective HCX PL APO CS 10.0x0.40 DRY UV at an extinction wavelength of 561 nm with 80% acousto-optic tunable filter (AOTF). Using overlapping optical sections, passed through the whole specimen with an ideal number of sections determined by the software, series of stacks were generated. The obtained images were finalised with maximum projection and Adobe Photoshop CS6 for adjusting colour, contrast, and brightness.

Descriptive terminology for body and appendage morphology was adopted from Lang (1948) and Huys and Boxshall (1991). Terminology referring to phylogenetic aspects follows Ax (1984); the terms “telson” and “furca” are adopted from Schminke (1976).

The English text was improved by using a free version of DeepL.

Abbreviations used in the text and figures: **A1** = antennule; **A2** = antenna; **aes** = aesthetasc; **ACST** = anterior cephalothoracic setulose tufts; **AST1– AST4** = abdominal lateral setulose tufts; **benp** = basoendopod; **CLVPa** = cephalothoracic anterior lateroventral processes; **CLVPp** = cephalothoracic posterior lateroventral processes; **CLDP** = cephalothoracic laterodorsal processes; **cphth** = cephalothorax; **DP(1–4)** = dorsal processes (1–4); **exp** ( **enp**) 1 (2, 3) = proximal (middle, distal) segment of exopod (endopod); **FLH** = cephalothoracic frontolateral horns; **FR** = furcal ramus/rami; **GDS** = genital double somite; **GF** = genital field; **GST** = lateral setulose tufts on female genital somite; **md** = mandible; **mx** = maxilla; **mxl** = maxillule; **mxp** = maxilliped; **P1– P6** = swimming legs 1–6; **RST** = rostral setulose tuft; **TP** = tube pore(s); **TST1– TST4** = lateral thoracic setulose tufts.

The type material is deposited in the Museu de Oceanografia Petrônio Alves Coelho – Universidade Federal de Pernambuco (**MOUFPE**), Brazil.

## ﻿Results

### ﻿Taxonomy

**Order Harpacticoida Sars, 1903**

**Family Cletodidae T. Scott, 1905**

**Subfamily Cletodinae T. Scott, 1905**

#### ﻿Genus Pseudechinopsyllus

Taxon classificationAnimaliaHarpacticoidaCletodidae

George, 2006

189AFC48-84BF-538B-8598-0EC533BF2F71

##### Generic diagnosis.

Cletodidae T. Scott, 1905, Cletodinae T. Scott, 1905, *Ceratonotus* group sensu Conroy-Dalton (2001). Body remarkably long and slender, female length from rostrum to end of FR between 1,100 and 1,400 µm, males somewhat smaller. Cphth frontally with strongly developed “peak”, with or without FLH, ACST present, and with four pairs of long tube pores. CLVPa and CLVPp well developed. Rostrum small, constricted and almost squarish or strongly elongated, apically with pair of sensilla, long tube pore, and RST. CLDP well developed or absent. Free thoracic somites bearing P2–P5 laterally with TST1–TST4, DP1–DP4 long, unarmed, with sensilla at their tips; dorsally and dorsoventrally bearing long tube pores. Females with last thoracic and first abdominal somite fused to GDS; former separation still indicated by dorsal suture; GST developed. GDS and remaining abdominal somites except telson also carrying dorsal and dorsoventral tube pores. Abdominal somites with or without AST1–AST3, whilst AST4 are missing in both known species. Telson at the most as large as the previous somite, laterally with two pairs of tube pores; anal operculum with dense row of fine long spinules at apical margin. FR very long and slender, reaching a length-width ratio between 10/1 and 14.8/1. All seven furcal setae present. Female A1 four-segmented, aes on third and fourth segment. Male A1 eight-segmented, haplocer. A2 lacking exopod, with allobasis bearing two abexopodal setae; endopod with two bipinnate spines and one small bare seta at anterior margin; apically with six elements, three (or 4) of which long and geniculate. Mouthparts overlapped by big labrum. Md with one-segmented palp, carrying basal seta no. 2 and endopodal setae nos. 3–5; setae no. 1 and no. 6 completely reduced. Mxl with distinct coxa that bears one apical seta; basis also separated, with two lateral and two or three apical setae. Mx with two endites and distinct allobasis; endopod distinct, small and knob-like, carrying two setae. Mxp with syncoxa and basis; both segments equipped with small and/or long spinules; syncoxa without seta; basis longer than syncoxa, outer margin convex; endopod produced into long, curved, and bare claw that is longer than the endopod and accompanied by a smaller bare seta. P1–P4 with transversely elongated bases that are covered by long spinules and carry a long tube pore at the anterior margin. Intercoxal sclerites transversely long, bow-like. Coxae small, trapezoid or rectangular in shape. P1 lacking endopod; basis with one outer bare or bipinnate and one inner bare seta that is located close to the coxa and arises from a protruded pedestal; exopod 2-segmented, exp1 with bipinnate outer spine, exp2 much longer than exp1, with one bipinnate outer spine and four geniculate apical setae; subapically with one tube pore. P2–P4 with three-segmented exopods; exp3 with 1:2:1 inner setae, respectively. P2 lacking endopod, female P3 and P4 each with one-segmented tiny endopod that bears one bare seta; male P3 and P4 endopods two-segmented, P3 enp2 additionally with long curved apophysis. Female P5 with basoendopod and fused or distinct exopod. Outer basoendopodal seta located on an extremely elongated and slender setophore, which is basally accompanied by one long tube pore. Endopodal lobe narrow but elongated, (sub)apically with two or three tube pores and two setae, one of which long and bipinnate, the other one small and bare. Exopod(al lobe) elongate, carrying two outer, two apical, and one subapical inner seta; additionally subapically with long tube pore. Male P5 with distinct endo- and exopod. Type species: *Pseudechinopsyllussindemarkae* George, 2006; additional species: *P.andrei* sp. nov. (present contribution).

#### ﻿ Pseudechinopsyllus andrei sp. nov.

Taxon classificationAnimaliaHarpacticoidaCletodidae

1DA1BC46-6426-5043-B1CF-AB9A215EBBE3

https://zoobank.org/A8715BE5-20F7-4E89-9CED-826540058FDA

##### Locus typicus.

Grussaí Canyon, Campos Basin, Brazil, geographic position 21°55'S, 39°50'W, 1,300 m depth.

##### Type material.

***Syntypes*** (ST) 1: female, put on 16 slides, MOUFPE collection no. 22056/1–16; ST2: female, put on 1 slide, MOUFPE collection no. 22057; ST3: female, put on 1 slide, MOUFPE collection no. 22058; ST4: male, put on 1 slide, MOUFPE collection no. 22059; ST5: male, put on 1 slide, MOUFPE collection no. 22060; ST6: female, put on 1 slide, MOUFPE collection no. 22061; ST7: female, put on 1 slide, MOUFPE collection no. 22062; ST8: female, put on 1 slide, MOUFPE collection no. 22063; ST9: female, put on 1 slide, MOUFPE collection no. 22064 (used for CLSM); ST10: male, put on 3 slides, MOUFPE collection no. 22065/1–3; ST11: copepodid CIV, put on 1 slide, MOUFPE collection no. 22066.

##### Note.

Because all representatives of the type material show damage to different parts of the body or appendages, the description was not based on one but on several individuals. The figure captions refer to the syntype on the basis of which an appendage or body section was drawn.

##### Description of female.

**Figure 2.**
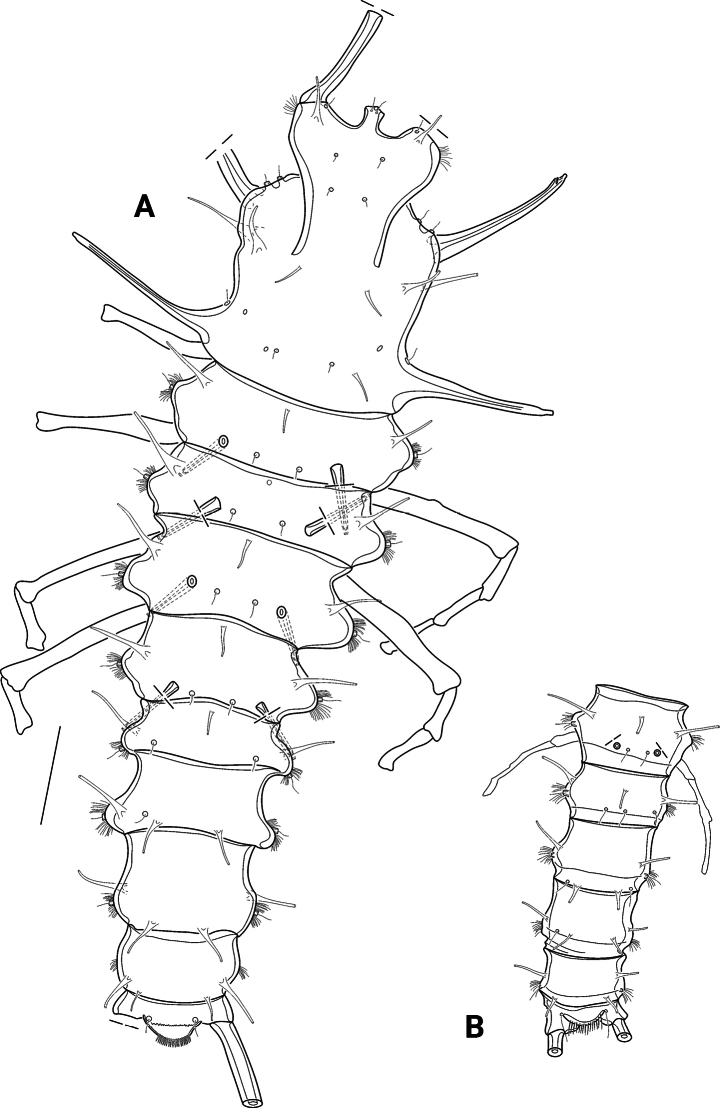
*Pseudechinopsyllusandrei* sp. nov. **A** female habitus, dorsal view (syntype 2) **B** male urosoma, dorsal view (syntype 5). Scale bar: 100 µm.

**Figure 3.**
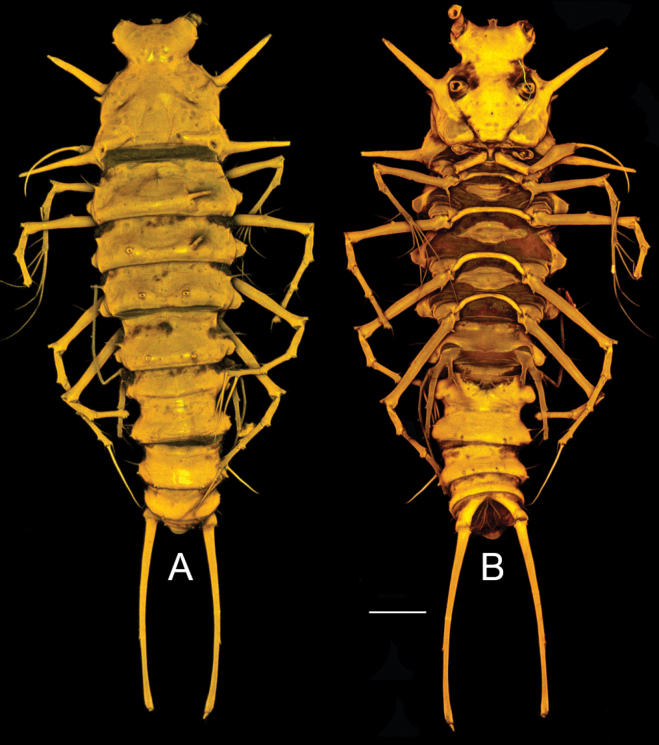
*Pseudechinopsyllusandrei* sp. nov., CLSM microphotograph, female habitus **A** dorsal view **B** ventral view (syntype 2). Scale bar: 100 µm.

**Figure 4.**
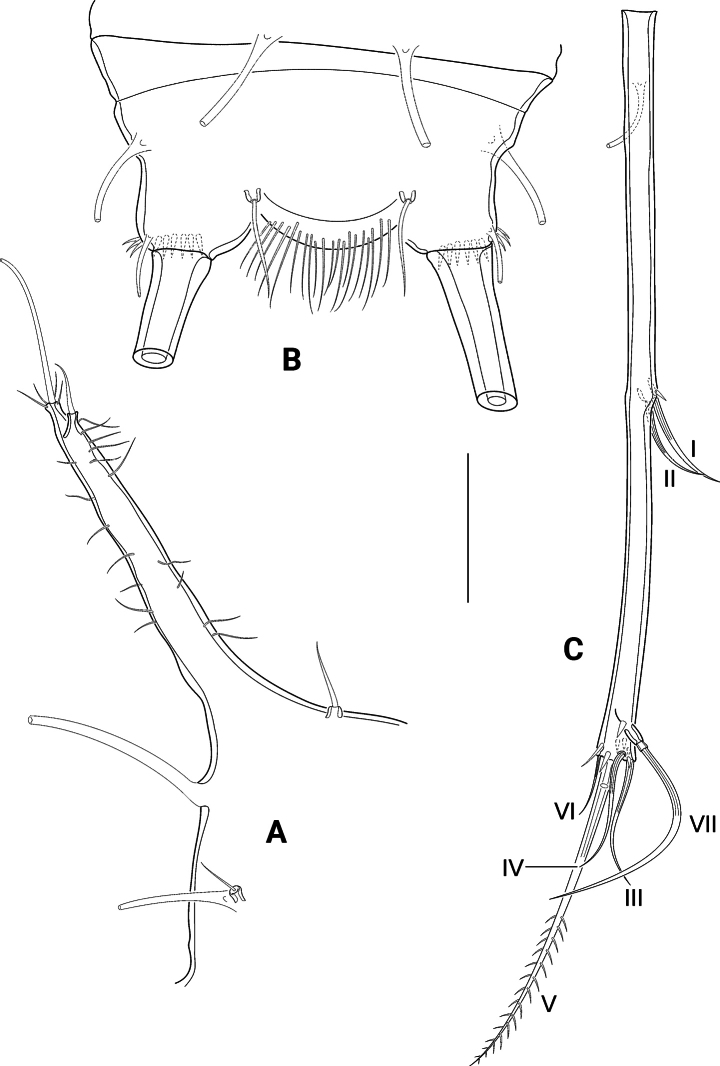
*Pseudechinopsyllusandrei* sp. nov. (syntype 1) **A** left anterior cephalothoracic lateroventral process (CLVPa), dorsal view **B** telson, dorsal view **C** right furcal ramus, dorsal view. Scale bar: 50 µm.

**Figure 5.**
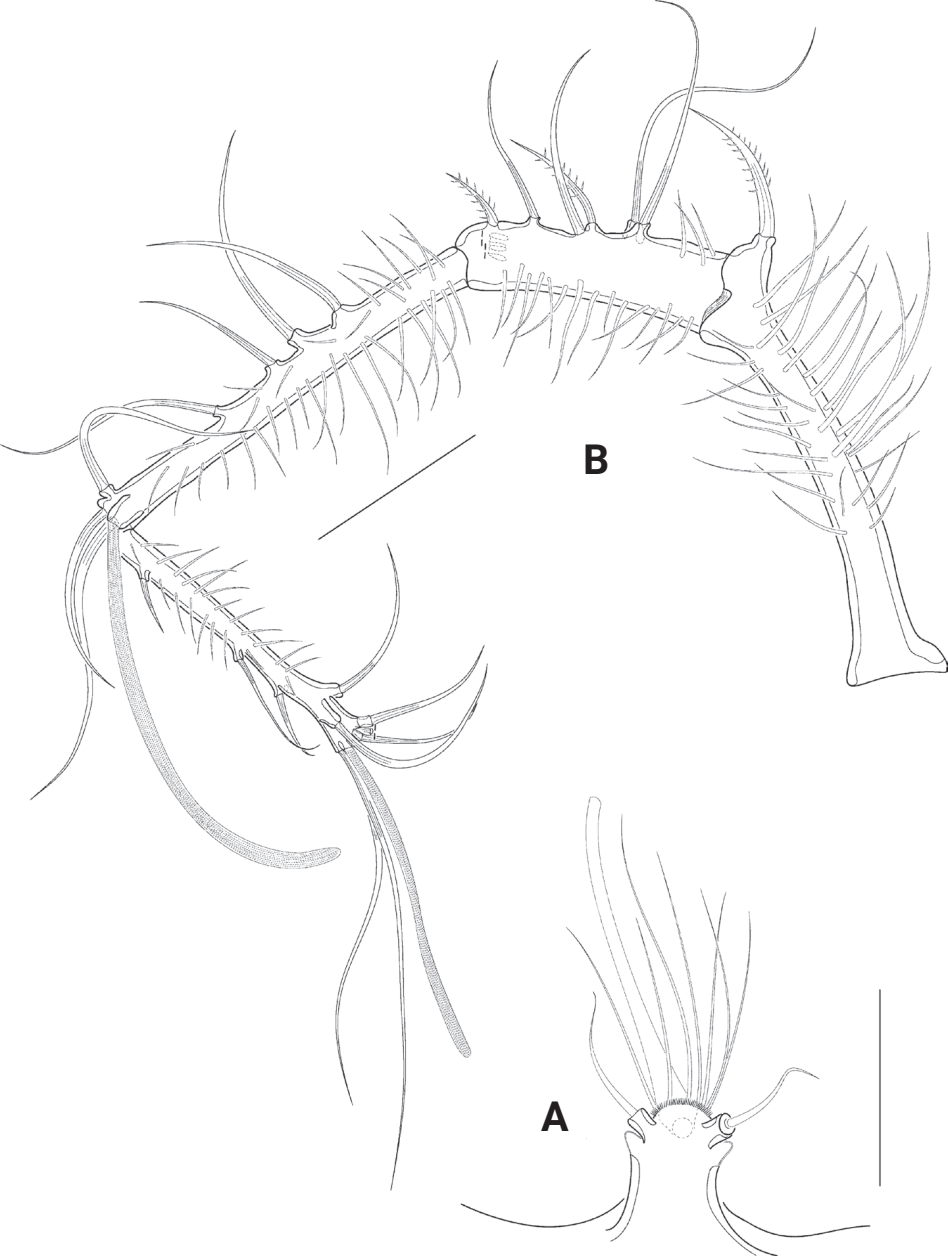
*Pseudechinopsyllusandrei* sp. nov. **A** rostrum, dorsal view (syntype 10) **B** female A1 (syntype 1). Scale bars: 50 µm.

Habitus (Figs 2A, 3A, B) long and slender, mean body length (R to end of FR) 1,255.9 µm (range 1,155.9–1,400.0 µm; *n* = 5). Cphth frontally with strongly developed peak, lacking FLH but bearing ACST, and four pairs of long tube pores. CLVPa (Figs 2A, 3A, B, 4A) and CLVPp (Figs 2A, 3A, B) well developed. Rostrum (exemplified in Fig. 5A) small, constricted and almost squarish, apically with pair of sensilla, long tube pore, and RST; previous to the sensillar pedestals, with hyaline outgrowths. CLDP absent. Free thoracic somites bearing P2–P5 laterally with TST1–TST4 and long, unarmed DP1–DP4 that carry sensilla at their tips; somites dorsally and dorsoventrally bearing long tube pores.

Females with last thoracic and first abdominal somite fused to GDS; former separation still indicated by dorsal suture; GST developed. GDS and remaining abdominal somites except telson also carrying dorsal and dorsoventral tube pores.

Abdominal somites with AST1–AST3, whilst AST4 are missing. Telson (Figs 2A, 3A, B, 4B) at the most as large as the previous somite, laterally with two pairs of tube pores; anal operculum with dense row of fine long spinules on apical margin. FR (Fig. 4C) reaching up to 1/3 of the body length, ~ 15× longer than wide, with long tube pore ventrally, close to proximal margin.

Seven setae present: I and II standing closely together laterally in the middle of FR, III subapically at the outer margin. IV, V and VI terminally, being IV as long as III, VI smaller; V longest and bipinnate. VII biarticulated, inserting dorsally.

A1 (Fig. 5B) four-segmented, all segments covered with long slender spinules. First, third, and fourth segments of nearly the same length, second segment somewhat shorter. First segment apically with one bipinnate seta. Second segment with eight setae (two broken and two bipinnate). Third segment bearing five bare setae at its anterior margin and an acrothek consisting of one aes and two bare setae. Fourth segment with ten bare setae (1 broken), of which two are fused at their bases and arising from a knob together with a small aes. Setal formula: I–1, II–8, III–5 (+ 2 and aes), IV–8 (+ 2 and aes).

**Figure 6.**
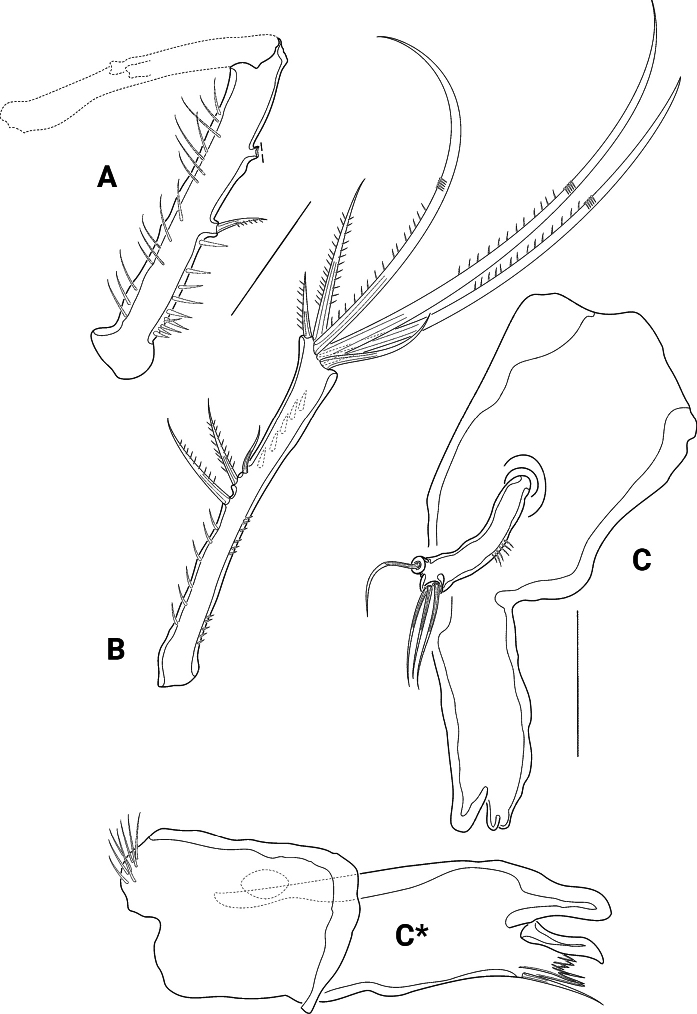
*Pseudechinopsyllusandrei* sp. nov. **A**A2 basis (syntype 10) **B**A2 endopod (syntype 1), C, C* Md, showing a semi-schematic contour of the gnathobase plus the detailed md palpus (**C**) and the detailed gnathobase of the counterpart (C*) (syntype 1). Scale bars: 50 µm.

A2 (Fig. 6A, B) lacking exp. Allobasis with two abexopodal setae (1 seta broken in Fig. 6A). Enp (Fig. 6B) anteriorly with row of spinules, medially with two pinnate and one smaller bare seta. Terminally with six setae, at least three of which long, unipinnate and geniculated, the anterior ones short and bipinnate, the posterior seta bare.

Md (Fig. 6C, C*) gnathobase strong, with two strongly sclerotised teeth that vary in their shape, one broad brush-like element and one small bare seta. Md palp (Fig. 6C) formed by fused basis, enp, and exp; long and slender, with basal seta no. 2 and endopodal setae nos. 3–5; basal seta no. 1 and exopodal seta no. 6 completely reduced.

**Figure 7.**
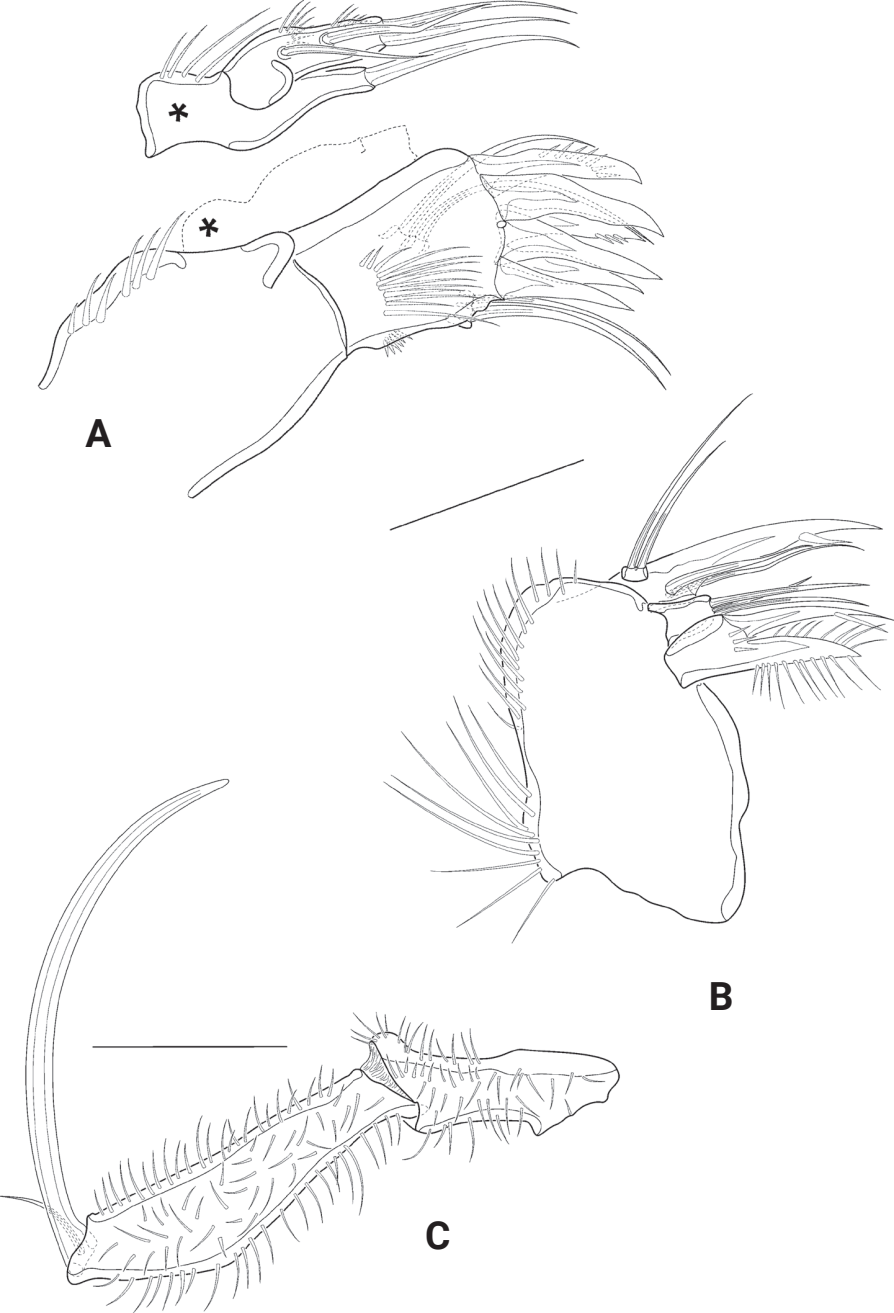
*Pseudechinopsyllusandrei* sp. nov. (syntype 1) **A** Mxl, coxa, and basis (marked with *) separated **B** Mx **C** Mxp. Scale bars: 50 µm.

Mxl (Fig. 7A) praecoxal arthrite basally with row of spinules; terminally with four bare and two unipinnate strong spines; subapically with two bare setae; additionally, with two bare surface setae, and on the other side with curved row of long spinules. Coxal endite with one bare small seta. Basis fused with enp and exp, forming a single segment that carries two lateral and two apical setae.

Mx (Fig. 7B) syncoxa with two distinct endites, few long spinules along outer basal edge, and row of spinules along other distal margin.

Proximal endite stronger than distal one, with few spinules and two spines, the proximal one biplumose and fused to the endite, the distal one with one single ruffle; the distal endite apically with three small bare setae. Allobasis distinct, produced into strong claw that carries one strong spinulose ruffle; additionally, with two bare setae. Endopod small and knob-like, equipped with two bare setae.

Mxp (Fig. 7C) prehensile, syncoxa and basis densely covered with long spinules, syncoxa lacking seta. Enp produced into long bare claw, accompanied by slender seta at its base.

**Figure 8.**
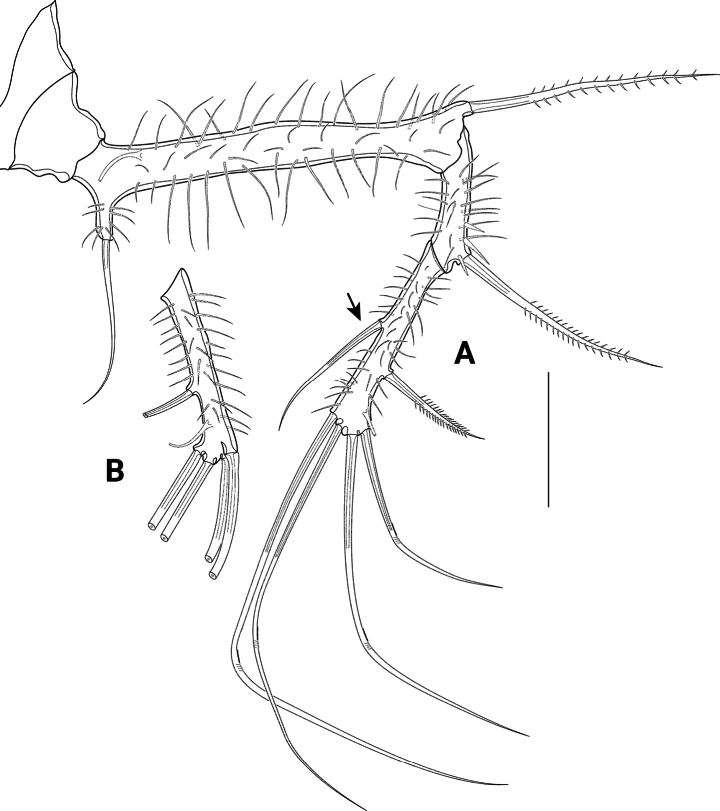
*Pseudechinopsyllusandrei* sp. nov. (syntype 1), P1**A** left leg, arrow indicating abnormal supernumerous seta on exp3 **B** Exp3 of right leg, showing the normal setation. Scale bar: 50 µm.

P1 (Fig. 8A, B) with transversely elongate basis and two-segmented exopod, both covered with long spinules; endopod absent.

Basis with one bipinnate outer and one bare inner seta, the latter arising from protruding pedestal. Tube pore inserting proximally close to anterior margin. Exp1 ~ 1/2 as long as exp2, with one bipinnate outer seta; exp2 with one bipinnate outer spine and four (sub)apical geniculate setae, which present a very fine serration previous to the geniculation. Subapically on anterior surface with tube pore.

*Pseudechinopsyllusandrei* sp. nov. exhibits a remarkable intraspecific variability that is expressed in the abnormal development of supernumerary setae or even segments on some swimming legs in single individuals. For example, the left P1 exp2 on syntype 1 bears an inner seta (arrow in Fig. 8A), which is missing on the right exp2 (Fig. 8B). We interpret this additional seta as abnormal because it is not present in any other species of the *Ceratonotus* group. Further abnormal setal and segment formation was found on the P2 and the P5 (see below).

**Figure 9.**
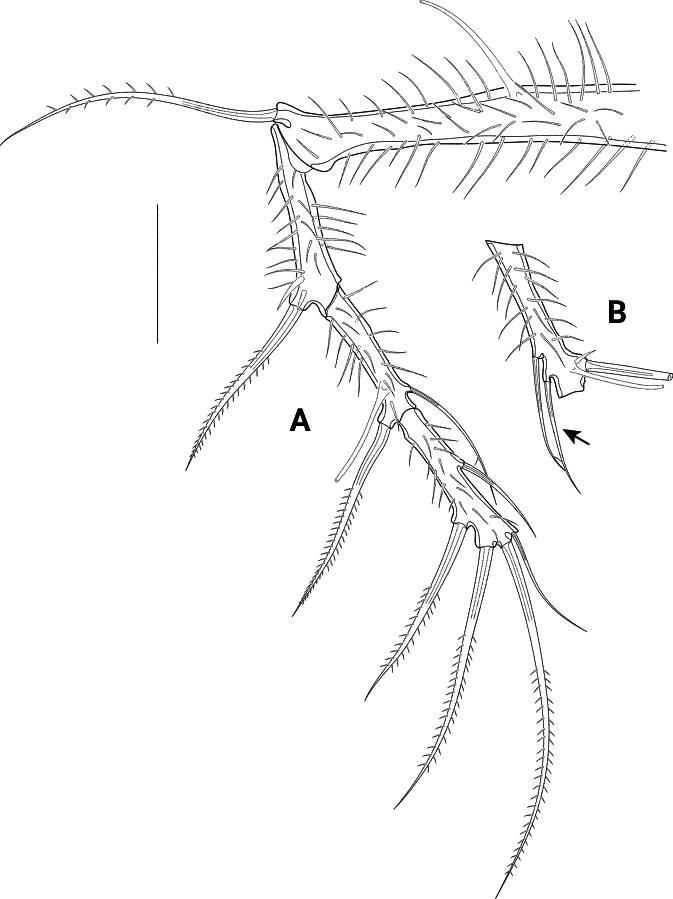
*Pseudechinopsyllusandrei* sp. nov. (syntype 1), P2 **A** right leg **B** Exp2 of left leg, arrow indicating abnormal supernumerous seta. Scale bar: 50 µm.

**Figure 10.**
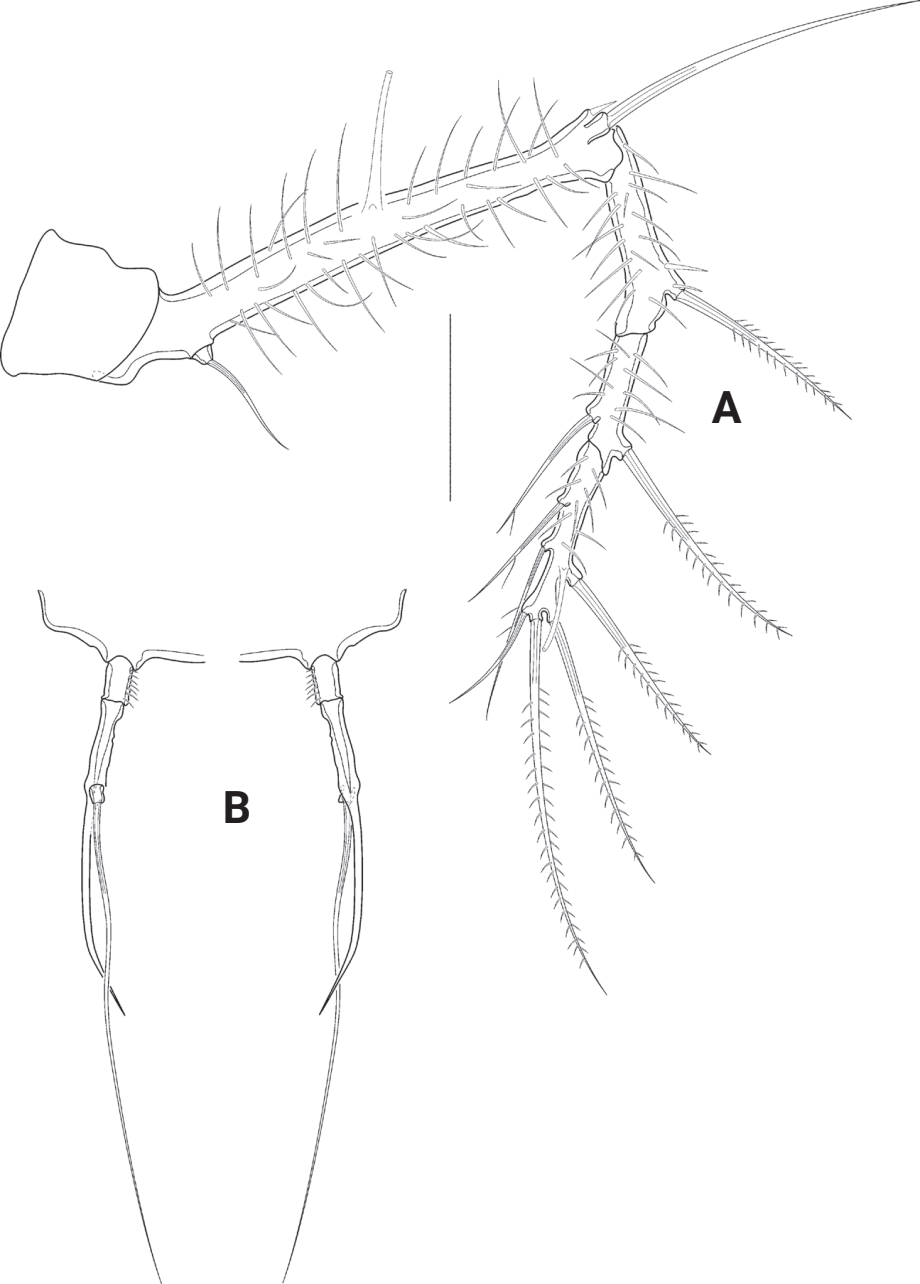
*Pseudechinopsyllusandrei* sp. nov. **A** female left P3 (syntype 1) **B** male P3 endopod, anterior (right-hand illustration) and posterior view (left-hand illustration) (syntype 10). Scale bar: 50 µm.

**Figure 11.**
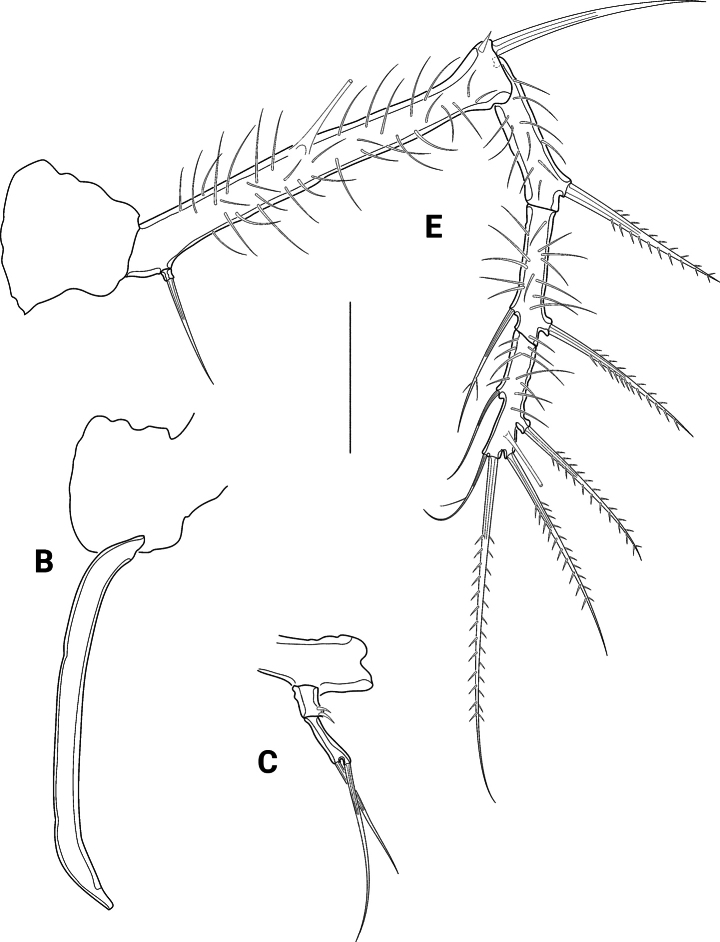
*Pseudechinopsyllusandrei* sp. nov. **A** female left P4 (syntype 1) **B** female P4 intercoxal sclerite (syntype 1), **C.** Male P4 right endopod (syntype 10). Scale bar: 50 µm.

P2–P4 (Figs 9A, B, 10A, 11A, B) with laterally elongated, bow-like intercoxal sclerites as exemplified for P4 (Fig. 11B) and transversely elongate bases that are covered with several long spinules and carry one outer seta (bipinnate in P2, bare in P3 and P4) and one very long tube pore. Exopods three-segmented; exp1 with one bipinnate outer seta, exp2 with one bare inner seta and one bipinnate outer spine; in P2 additionally with long tube pore. In Syntype 1, however, like the left-hand P1 exp2 also the left-hand P2 exp2 bears an additional inner seta (arrow in Fig. 9B). Exp3 with two outer spines and two apical setae, the innermost of which narrower and shorter than the outermost one; P2 and P4 exp3 additionally with one inner, P3 exp3 with two inner setae. P3 laterally, P4 subapically at outer margin with tube pore. P2 (Fig. 9A) lacking enp. P3 and P4 (Figs 10A, 11A) each with small knob-like enp carrying one bare seta. Setal formula shown in Table 1.

**Table 1.** *Pseudechinopsyllusandrei* sp. nov., setation of swimming legs P2–P4. Roman numerals indicate outer setae/spines.

	Exp1	Exp2	Exp3	Enp1
P2	I;0	I;1	II;2;1	–
P3	I;0	I;1	II;2;2	1
P4	I;0	I;1	II;2;1	1

**Figure 12.**
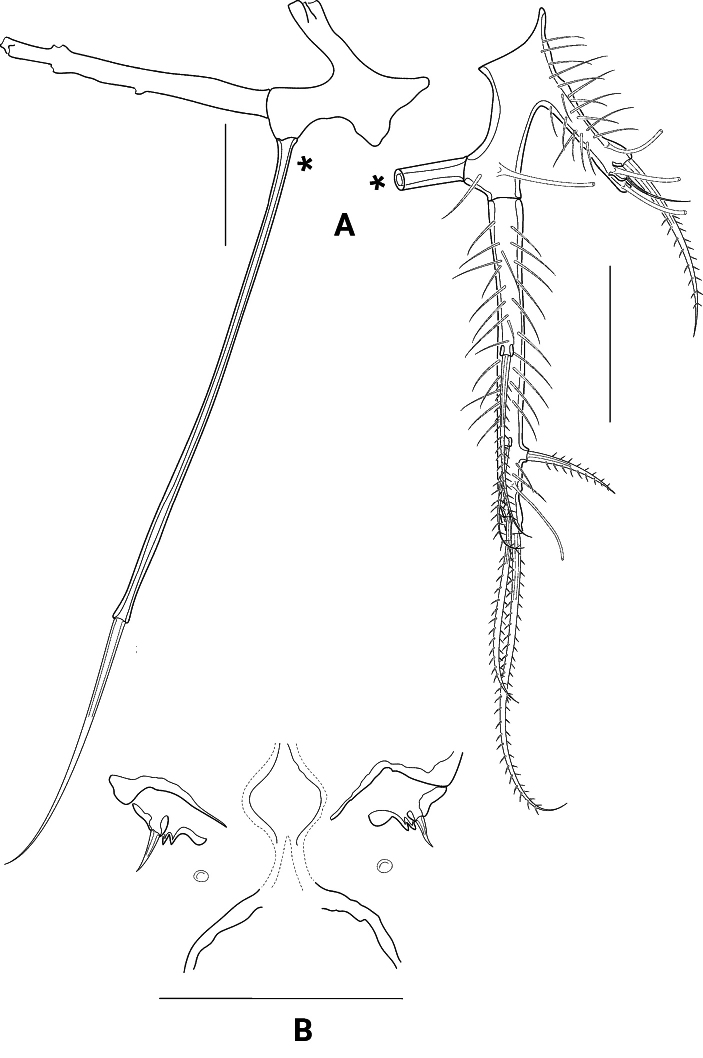
*Pseudechinopsyllusandrei* sp. nov. **A** female right P5; * showing the excessively long setophore (syntype 1) **B** female genital field with rudimentary P6 (syntype 7). Scale bars: 50 µm.

P5 (Fig. 12A) Basoendopod and exopod not fused. Endopodal lobe of basoendopod with long spinules, one tiny bare and one long bipinnate seta, accompanied by three tube pores. Outer basoendopodal seta arising from extremely long setophore (see asterisk*), which is almost twice the length of the exopod and accompanied by one tube pore. Exp also equipped with long spinules, and with two outer, one subapical, one apical and one inner bipinnate setae, as well as with long slender tube pore distally.

GF (Fig. 12B) P6 strongly reduced in size, each lobe bearing one tiny bare apical seta; margin on the inner side of the setae grown out into two small but distinctive spikes. Gonopore only indistinctly recognisable, ovoid in shape, and opening into two distal sclerotised formations.

**Male.** The male resembles the female in most characters, with the exception of the (sexually dimorphic) body size, development of the segments between thorax and abdomen, and in the likewise sexually dimorphic shapes of the A1, the P3 and P4 endopods, the P5, and the P6.

Habitus almost identical to that of the female but clearly (up to 1/3) smaller (mean 944.6 µm; range 908.8 µm–979.4 µm; *n* = 3) and lacking GDS (cf. Fig. 2B).

**Figure 13.**
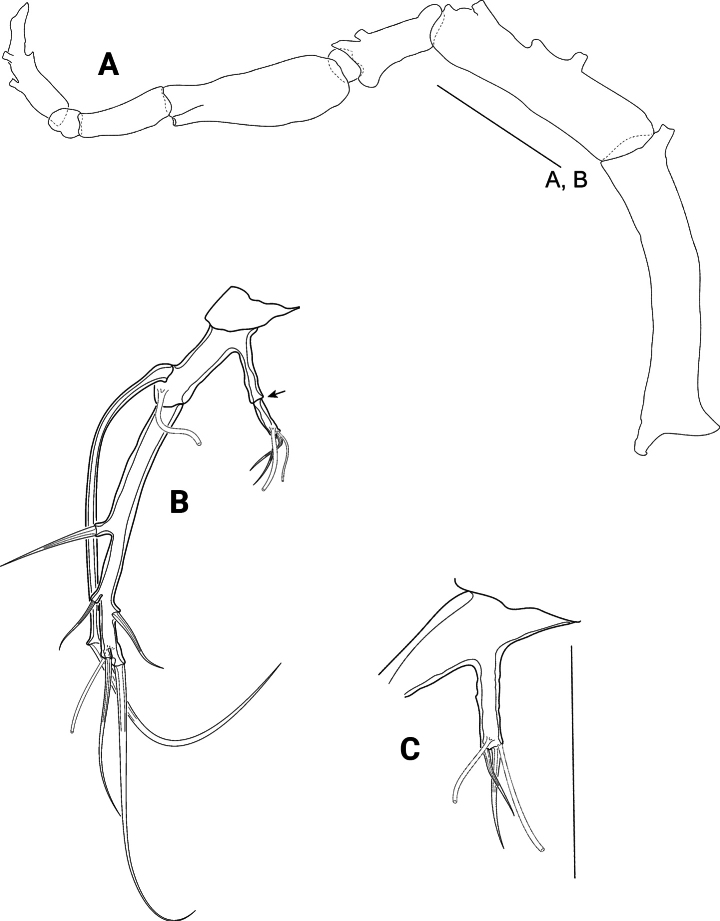
*Pseudechinopsyllusandrei* sp. nov., male **A** contour of the A1 (syntype 10) **B** P5, arrow pointing to the abnormally formed segment boundary (syntype 10) **C** P5 basoendopod, normal shape: endopodal lobe extended but 1-segmented. Scale bars: 50 µm.

**Figure 14.**
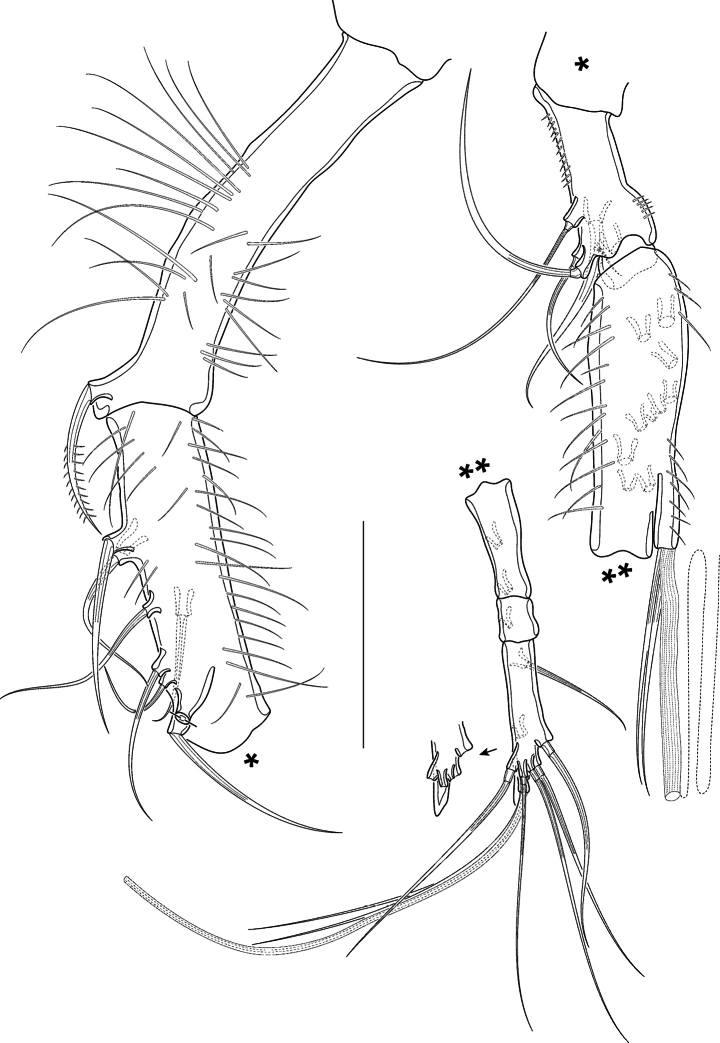
*Pseudechinopsyllusandrei* sp. nov., male A1 (syntype 10) **A** first and second segments **B** third to fifth segments **C** sixth to eighth segments; single (*) and double asterisks (**) indicate the respective connections of the segments; length of the aes of the fifth segment indicated by dotted line; spatulate tip of eighth segment accentuated by arrow. Scale bar: 50 µm.

A1 (Figs 13A, 14) eight-segmented, haplocer. First segment longest, with several long spinules, and with one bipinnate distal seta (Fig. 14); second segment smaller than first one, with eight bare setae and several long spinules posteriorly (Fig. 14); third segment much smaller than preceding segments, with five bare setae (Fig. 14); fourth segment smallest, no setae discernible (Fig. 14). Fifth segment very slightly swollen, with rows of spinules, and bearing ten setae (all broken in Fig. 14) plus acrothek formed by two setae and one aes arising from long protrusion; sixth segment slender, with two setae (broken in Fig. 14); seventh segment small, with one seta (broken in Fig. 14); eighth segment enlarged, distally acute, with seven bare setae (1 broken in Fig. 14) and a subapical acrothek formed by aes and two additional setae. Segment apically spatulate (arrowed in Fig. 14). Setal formula: I-1; II-8; III-5; IV-0; V-12 + aes; VI-2; VII-1; VIII-9 + aes.

P3 exopod as in female; endopod (Fig. 10B) three-segmented, enp1 small, with some spinules at outer margin; enp2 longest segment, with a slender outwardly curved apophysis that is longer than the whole endopod; enp3 minute, with one long bare apical seta.

P4 exopod as in female; endopod (Fig. 11C) small, two-segmented, enp1 with few spinules on inner margin, enp2 with two bare apical setae.

P5 (Fig. 13B, C) of similar shape as that of female but without spinulose coverage. Endopod also fused to the basis (= basoendopod), exopod distinct. The outer basal setophore (Fig. 13B) is shorter than in the female, only reaching the length of the exopod. Endopodal lobe (Fig. 13C) of syntypes 4 and 5 apically with two short bare setae and two tube pores. In contrast, the endopodal lobe of syntype 10 is divided into two segments (Fig. 13B; arrow indicating boundary), armouring of second segment as in syntypes 4 and 5.

P6 not even rudimentarily recognisable, apparently completely reduced.

##### Etymology.

The epithet *andrei* is given in fondly and grateful memory of Prof. Dr André M. Esteves (Federal University of Pernambuco (UFPE), Brazil), who passed away on 30 April 2025.

### ﻿Phylogenetic analysis

The aim of the phylogenetic analysis was threefold:

- Justification of *Pseudechinopsyllus* as a monophylum and assignment of *P.andrei* sp. nov. to that genus;
- Characterisation of *P.sindemarkae* and *P.andrei* sp. nov. as independent species;
- Examination of a closer relationship between *Pseudechinopsyllus* and *Echinopsyllus*.

*Pseudechinopsyllus* and *Echinopsyllus* undoubtedly belong to the *Ceratonotus* group (George 2020, 2021), which is why no corresponding further phylogenetic justification is provided. It is also not the intention of the present study to provide a comprehensive phylogeny of the *Ceratonotus* group itself. The selection of characters for the analysis conducted here is therefore limited to 41 potential aut- and synapomorphies in the direct comparison of *Pseudechinopsyllus* and *Echinopsyllus*. The characters are listed in Table 2.

**Table 2.** List of morphological characters used in the provided phylogenetic analysis. Plesiomorphic character states are set in square brackets in the second column; apomorphic character states are marked with a “1”; apomorphic characters that may occur convergently in some other *Ceratonotus* group taxa are shown in bold type.

No.	Character/taxon	*Echinopsyllus*	*P.sindemarkae*	*P.andrei* sp. nov.
**1**	**Cphth forehead developed to prominent peak [peak at the most moderately developed**]		**1**	**1**
2	P2 basis densely covered with long spinules [basis without dense coverage]		1	1
3	P3 basis densely covered with long spinules [basis without dense coverage]		1	1
4	P4 basis densely covered with long spinules [basis without dense coverage]		1	1
5	P2 exp1 densely covered with long spinules [spinules at most on outer and inner margin]		1	1
6	P2 exp-2 female TP remarkably elongated [TP small]		1	1
**7**	**Rostrum in front of the sensilla base strongly elongated [not elongated**]		**1**	
8	FR proximal part studded with spinules [FR with at most single spinules]		1	
9	Lateral TP on cphth excessively elongated [moderately elongated]		1	
10	2^nd^ abdominal somite dorsally with pair of double cuticular cusps [cusps absent]		1	
11	P5 female exopodal part with excessively long subapical tube pore [tube pore short]		1	
12	P5 female tube pore near setophore extraordinarily long [tube pore short]		1	
13	FLH secondarily lost [FLH still present]			1
14	CLDP secondarily lost [CLDP still present]			1
15	A1 female 3^rd^ segment densely covered with long spinules [segment at most bearing few long spinules]			1
16	A1 female 4^th^ segment densely covered with long spinules [segment unarmoured]			1
17	P1 insertion base of inner basal seta remarkably protruded [only tiny pedestal]			1
18	P5 female exopod(al lobe) 10× longer than broad [at most 6× longer than broad]			1
19	P5 setophore twice as long as exopod [at most slightly longer than exopod]			1
20	Rostral sensilla branched [sensilla bare]	1		
21	CADP developed [no CADP] (character 1 in Wandeness et al. 2009)	1		
22	Bulbous projection between CADP and CLDP [bulbous projection not developed] (character 2 in Wandeness et al. 2009)	1		
23	CAPD and CLDP with long hair-like setules [no such setules present] (character 3 in Wandeness et al. 2009)	1		
24	Sensilla on cphth and body somites branched [sensilla of simple shape] (character 4 in Wandeness et al. 2009)	1		
25	DT2 transformed into V-shaped processes [at most formed as tubercles] (character 6 in Wandeness et al. 2009)	1		
26	DT3 transformed into V-shaped processes [at most formed as tubercles] (character 7 in Wandeness et al. 2009)	1		
27	A1 female penultimate segment with at most 5 setae [with ≥ 6 setae]	1		
28	FR elongated between seta VII and apical margin, shifting seta VII mid-length [seta VII subapically] (character 8 in Wandeness et al. 2009)	1		
29	FR seta VII: first article remarkably elongated [article short]	1		
30	P5 seta 5 lost [still present] (character 5 in Wandeness et al. 2009)	1		
31	Female body length > 900 µm [female body length < 750 µm]	1	1	1
32	CLVPa long, rigid, sclerotised [CLVPa smooth, flexible] (character 29 in Wandeness et al. 2009)	1	1	1
33	CLVPa basal TP displaced (sub)apically [TP still inserting basally] (character 30 in Wandeness et al. 2009)	1	1	1
34	CLVPp long, rigid, sclerotised [CLVPa smooth, somewhat flexible] (character 31 in Wandeness et al. 2009)	1	1	1
**35**	**DP4 developed [no DP4**]	**1**	**1**	**1**
**36**	**Md palp seta 1 lost [still present] (character 33 (part) in Wandeness et al. 2009)**	**1**	**1**	**1**
**37**	**Md palp seta 6 lost [still present] (character 33 (part) in Wandeness et al. 2009)**	**1**	**1**	**1**
**38**	**Mxl coxa with 1 seta/spine [with at least 2 elements] (character 34 in Wandeness et al. 2009)**	**1**	**1**	**1**
39	Mxp syncoxa without seta [seta present]	1	1	1
40	P2 exp2 with TP [no TP developed]	1	1	1
41	P5 setophore at least as long as exopod [setophore small]	1	1	1

### ﻿Justification of *Pseudechinopsyllus* as a monophylum and assignment of *P.andrei* sp. nov. to that genus

The comparison of *P.sindemarkae* and *P.andrei* sp. nov. yielded six derived characters (Table 2, characters 1–6), all except one exclusively shared by these two species, which could be proven by means of a comparison with the remaining taxa of the *Ceratonotus* group. They are postulated to be synapomorphies of *P.sindemarkae* and *P.andrei* sp. nov. Based on these, the monophyly of *Pseudechinopsyllus* can be satisfactorily and unequivocally substantiated.

### ﻿Characterisation of *P.sindemarkae* and *P.andrei* sp. nov. as independent species

Both *P.sindemarkae* and *P.andrei* sp. nov. can be characterised as independent species by a number of exclusive derived characters. For *P.sindemarkae*, six putative autapomorphies are listed in Table 2 (characters 7–12). Five of them are neither detectable in *P.andrei* sp. nov. nor in any other representative of the *Ceratonotus* group and thus characterise *P.sindemarkae* as an independent species.

In addition to the absence of characters 7–12, *P.andrei* sp. nov. can also be justified as a separate species by seven exclusive characters (Table 2: characters 13–19). None of them converges with *P.sindemarkae*. The apomorphic state of characters 13 and 14 are considered here as a further development, namely as a secondary reduction of the FLH and the CLDP instead of their primary absence, as assumed for *Paratouphapleura* and *Touphapleura* (for *Dorsiceratus* the judgement is still unclear, but does not play a role in the present context). Why we assumed a secondary reduction of these processes in *P.andrei* sp. nov. is explained in detail in the following discussion.

### ﻿Examination of a closer relationship between *Pseudechinopsyllus* and *Echinopsyllus*

Conroy-Dalton (2003a), in the frame of a detailed re-description of *Echinopsyllusnormani* Sars, 1909, assumed that this species holds an isolated position within the then Ancorabolinae. George (2006a) supposed that *Echinopsyllus* and *Pseudechinopsyllus* may be closely related on the basis of character similarities that, however, he had not examined in detail. Wandeness et al. (2009) carried out a comprehensive phylogenetic analysis, by means of which they attempted to substantiate the close relationship of the two genera on the basis of nine synapomorphies. Six of these were included in our analysis and are listed in Table 2 (characters 32–34, 36–38). However, one character mentioned by Wandeness et al. (2009: 74, character 32: “Rostrum elongate in its proximal half”) is not included because we cannot beyond doubt confirm an elongation of the proximal half of the rostrum for *Echinopsyllus*. In the case of *Pseudechinopsyllusandrei* sp. nov., however, it is clear that the rostrum was not extended; only *P.sindemarkae* shows a clear elongation of the proximal half (Table 2, character 7). Two further characters of Wandeness et al. (2009) were not considered because they are widespread in the *Ceratonotus* group, so that their assessment as synapomorphies is currently not possible.

In addition to the six synapomorphies mentioned by Wandeness et al. (2009), we found five more (Table 2, characters 31, 35, 39–41). The synapomorphies found are treated in detail in the discussion. They justify the close relationship of *Echinopsyllus* and *Pseudechinopsyllus* clearly and beyond doubt. Moreover, *Echinopsyllus* can also be clearly established as a monophylum by including the eight autapomorphies listed by Wandeness et al. (2009) and three additional putative autapomorphies (Table 2, characters 20–30).

### ﻿Key to the species of *Pseudechinopsyllus* George, 2006

**Table:** 

1	Rostrum long, clearly protruding over the frontal corners of the cphth; cephalothoracic laterodorsal processes (CLDP) long; cephalothoracic posterior lateroventral processes (CLVPp) anteriorly and posteriorly covered with spinules; female P5 exopod fused with basoendopod; endopodal lobe ~ 1.5× as long as wide; basal tube pore as long as exopodal lobe	***Pseudechinopsyllussindemarkae* George, 2006**
–	Rostrum short, not extending beyond the frontal corners of the cphth; CLDP absent; CLVPp bare; female P5 exopod and basoendopod separate; endopodal lobe almost 3× as long as wide; basal tube pore only ~ 1/3 the length of exopod	***Pseudechinopsyllusandrei* sp. nov.**

## ﻿Discussion

The discovery and description of a new species that can be assigned to a previously monotypic genus usually opens up the possibility for phylogenetic studies to corroborate that genus as a monophylum and its phylogenetic relationship to closely related taxa.

George (2020) confirmed the *Ceratonotus* group as a well-founded monophylum based on and extending Conroy-Dalton’s (2001) first comprehensive study. At that time, the group had grown to 29 species in nine genera (George 2020). A year later, George (2021) added four more genera, namely *Paratouphapleura* George, 2021, *Poropsyllus* George, 2021, *Pseudopolyascophorus*George 2021, and *Tauroceratus*George 2021, and described two species, bringing the *Ceratonotus* group to 31 species in 13 genera. Recently, George et al. (2025) carried out a comprehensive morphological study of the taxon *Dorsiceratus* and described three new species in this context. So, with *Pseudechinopsyllusandrei* sp. nov. described in the present work, the *Ceratonotus* group now comprises 35 species in 13 genera.

*Pseudechinopsyllussindemarkae* was described by George (2006a) based on one female from station #565 (water depth 403 m) on the north-eastern, and one female from station #511 (water depth 597 m) on the southern slope of the Great Meteor Seamount (GMS), a huge submarine elevation in the subtropical north-eastern mid-Atlantic (cf. George and Schminke 2002: fig. 1, tab. 4). Apart from two females and two copepodids of *Dorsiceratusursulae* George, 2006, the two female specimens found were the only representatives of the Ancorabolinae Sars, 1909 at the GMS, to which the *Ceratonotus* group sensu Conroy-Dalton (2001) also belonged at that time. Despite the possibility of a clear assignment to this group based on e.g., the armouring of cphth and body somites with cuticular processes, the lack of an exopod on A2, the strong transverse elongation of the bases of the swimming legs P1–P4 and other features, as well as a closer relationship to *Echinopsyllus* Sars, 1909 as assumed by George (2006a), the two females showed remarkable differences to *Echinopsyllus* as well as to the other representatives of the *Ceratonotus* group. Thus, George (2006a) felt compelled to place them in a new genus established for this purpose.

It was not the aim of this study to make a comprehensive contribution to the clarification of the phylogenetic relationships within the *Ceratonotus* group; this is the subject of an ongoing analysis. In the following, however, (A) the assignment of *P.andrei* sp. nov. to *Pseudechinopsyllus* and (B) the monophyly of the genus were phylogenetically justified. In addition, we examined (C) whether the hypothesis of Wandeness et al. (2009) of a close relationship of *Pseudechinopsyllus* and *Echinopsyllus* – which Lee and Huys (2019) viewed with a certain degree of scepticism – could be substantiated based on further morphological evidence.

### ﻿Justification of *Pseudechinopsyllus* as a monophylum and assignment of *P.andrei* sp. nov. to that genus

*Pseudechinopsyllussindemarkae* and *P.andrei* sp. nov. could be grouped into a monophylum based on six synapomorphies (Table 2, characters 1–6):

#### ﻿Character 1, cephalothoracic peak particularly prominent:

George’s (2006a) statement that character 1 is a unique feature of *P.sindemarkae* cannot be maintained. Representatives of *Dendropsyllus* Conroy-Dalton, 2003 and *Tauroceratus* George, 2021, as well as *Poropsyllusmenzelae* George, 2021 also exhibit prominent peaks (cf. George and Schminke 1998; Conroy-Dalton 2003b; George 2006b, 2021). Such a strongly protruding peak can be well justified as derived, because several representatives of the *Ceratonotus* group (*Dimorphipodia* Lee & Huys, 2019, *Paratouphapleura* George, 2021, *Touphapleura*) show only a slightly, others (*Arthuricornua* Conroy-Dalton, 2001, *Dorsiceratus*, *Polyascophorus* George, 1998, *Pseudopolyascophorus* (George, Wandeness & Santos, 2013)) a moderately developed peak (George 1998, 2006a, 2021; George and Schminke 1998; Conroy-Dalton 2001; George et al. 2013; Lee and Huys 2019). Moreover, in *Cletodes* Brady, 1872, the supposed sister taxon of the *Ceratonotus* group (cf. George 2020), such a peak is not developed at all. The question then arose as to whether the derived prominent peak can be hypothesised as a synapomorphy of the taxa mentioned, or whether it was developed convergently several times. We favoured the latter assumption, because according to George (2021) the taxa in question, *Dendropsyllus*, *Tauroceratus* and *Poropsyllus*, can each be very clearly characterised as independent monophyla by a number of autapomorphies, and *Pseudechinopsyllus* does not share any of them. As will be shown below, *Pseudechinopsyllus* in turn can be characterised by derivations that are not observed in the above-mentioned three taxa. Just like *P.sindemarkae*, however, *P.andrei* sp. nov. also has a particularly prominent peak.

In contrast, *Echinopsyllus* is characterised by a completely different frontal area of the cphth. As Sars (1909), Conroy-Dalton (2003a) and Wandeness et al. (2009) show for the species known to date, *Echinopsyllus* cannot be said to have a pronounced peak. In addition to the posterior CLDP common within the *Ceratonotus* group, the species of *Echinopsyllus* bear large, backwards directed and convexly curved appendages (CADP) in the anterior region of the cphth (Sars 1909; Conroy-Dalton 2003a; Wandeness et al. 2009), which are not found in any other representative of the *Ceratonotus* group (Table 2, character 35). Together with the CLDP and the “bulb” in between, the CAPD form a highly complex dorsal surface structure of the cphth (Sars 1909; Conroy-Dalton 2003a; Wandeness et al. 2009), which is particularly recognisable in lateral view and is not only unique within the *Ceratonotus* group but is unparalleled in the Harpacticoida as a whole. It represents a high-quality autapomorphy of the genus (Table 2, characters 21–23).

Due to the heterogeneous occurrence of a derived prominent peak in the *Ceratonotus* group, which is therefore to be considered a convergent development in our view, we considered its development to be an autapomorphy of *Pseudechinopsyllus*, which at the same time has been developed several times convergently within the *Ceratonotus* group. In Table 2, this character is listed as no. 1.

#### ﻿Characters 2–4, P2–P4 bases densely covered with long spinules:

The vast majority of taxa in the *Ceratonotus* group are characterised by a row of long spinules on the anterior margin of the swimming leg bases P2–P4 (e.g., *Touphapleura*, *Dorsiceratus* (part.); see George 1998, 2006a); single short or long spinules often also occur on the posterior margin of the basis (e.g., *E.normani*; see Conroy-Dalton 2003a), but they can also be completely absent (e.g., *Poropsyllusmenzelae* George, 2021). Occasionally, however, the basis of at least single swimming legs is covered with shorter or longer spinules, as for example in *Dimorphipodiachangi* Lee & Huys, 2019, *E.andrei* Wandeness, George & Santos, 2009, *E.nogueirae* Wandeness, George & Santos, 2009, and *E.grohmannae* Wandeness, George & Santos, 2009 (see Wandeness et al. 2009). However, only the P2–P4 bases of *Pseudechinopsyllussindemarkae* and *P.andrei* sp. nov. are extensively furnished with very long spinules (Figs 8A, 9A, 10A, 11A; George 2006a: fig. 21A–D), which gives them a truly “cactus-like” appearance. The extensive armouring with long spinules on these swimming leg bases was assumed here as a synapomorphy for *P.sindemarkae* and *P.andrei* sp. nov.

#### ﻿Character 5, P2 exp1 densely covered with long spinules:

*Pseudechinopsyllussindemarkae* and *P.andrei* sp. nov. show, like for the bases of their swimming legs P2–P4, an identical spinulose coverage on the first segment of the P2. This derivation is also considered as synapomorphic for both species.

#### ﻿Character 6, P2 exp-2 female tube pore remarkably elongated:

A significant similarity between *Echinopsyllus* and *Pseudechinopsyllus* is the presence of a tube pore on the second segment of the P2 exopod (see below, character 40); no other taxon in the *Ceratonotus* group has a tube pore at this location. Furthermore, this tube pore is extremely elongated only in *P.sindemarkae* and *P.andrei* sp. nov. (Table 2, character 6), whereas it remains small in all *Echinopsyllus* species. Thus, the strong elongation of the tube pore is assumed to be synapomorphic for the two *Pseudechinopsyllus* species.

The derived states of characters 1–6 allowed an unambiguous justification of a monophylum *Pseudechinopsyllus* with the type species *P.sindemarkae* established by George (2006a).

### ﻿Characterisation of *P.sindemarkae* and *P.andrei* sp. nov. as independent species

*P.sindemarkae* can be characterised as an independent species by means of six autapomorphies (Table 2, characters 7–12). All of them – the clearly elongated anterior rostral part (character 7), the FR studded with many spinules on their proximal section (character 8) the extremely elongated lateral tube pores on the cphth (character 9), the paired small cusps on the second abdominal somite (character 10), as well as the female P5 presenting an excessively long tube pore close to the setophore (Character 11) and subapically on the exopodal lobe (character 12) – occur exclusively in *P.sindemarkae*, substantiating its establishment as distinct species.

While George (2006a) thought to recognise the elongated anterior rostral region (character 7) in *P.sindemarkae* as a special generic feature, Wandeness et al. (2009: character 32) even assumed it to be a synapomorphy of *Pseudechinopsyllus* and *Echinopsyllus*. Obviously, both were wrong because *P.andrei* sp. nov. has a small, almost square rostrum (cf. Fig. 5A), which is more similar to those of the genus *Dorsiceratus* (Drzycimski 1967; Coull 1973; George 2006a; George and Plum 2009; George et al. 2025). And as noted above in relation to *Echinopsyllus*, we could confirm a clear elongation of the anterior rostral region for the species of this genus, as claimed by Wandeness et al. (2009). Therefore, such rostral elongation cannot be maintained neither as a generic autapomorphy for *Pseudechinopsyllus* nor a synapomorphy for that genus and *Echinopsyllus*. It must therefore be assumed that the long rostrum of *P.sindemarkae* is an exclusive new formation of this species.

On the other hand, it should not be concealed that, in addition to *P.sindemarkae*, with *Pseudopolyascophorusmonoceratus* (George, Wandeness & Santos, 2013) another representative of the *Ceratonotus* group bears a clearly elongated anterior rostral region (George et al. 2013: fig. 3A). However, both species differ so drastically from each other morphologically that an inheritance of the elongated rostrum from an ancestor common only to them seems unfounded. Apart from the matching elongation, the rostra themselves are also different in their shape. We therefore assumed a convergent development here.

**Figure 15.**
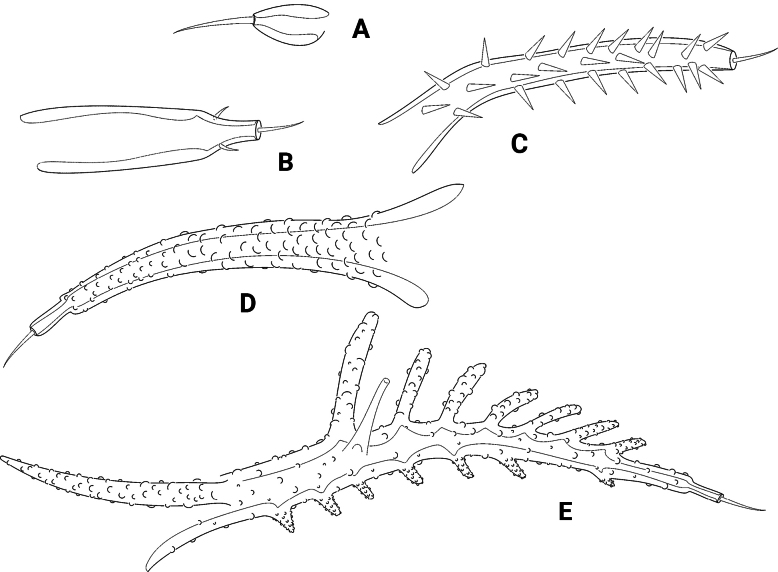
Semi-schematic illustrations of the thoracal dorsal processes 1 (DP1) of different representatives of the *Ceratonotus* group, which can be divided into five groups according to their length, structure, and armouring **A** group (i) **B** group (ii) **C** group (iii) **D** group (iv) **E** group (v), drawn to approximately the same scale. Further explanations in the text.

*Pseudechinopsyllusandrei* sp. nov. for its part is characterised by seven autapomorphies (Table 2, characters 13–19). Except for characters 13 and 14, all derived states occur exclusively in this species; they include the formation of particularly long spinules on segments 3 and 4 of the A1 (characters 15, 16), the formation of a strongly protruding attachment of the inner seta at the P1 basis (character 17) and the extreme elongation of the female P5 exopod (character 18) and of the setophore on the same swimming leg (character 19). All these characters are not found in any other taxon of the *Ceratonotus* group and undoubtedly represent autapomorphies of *P.andrei* sp. nov. However, some uncertainties exist with regard to characters 13 and 14, i.e., the here-presumed secondary loss of the frontolateral horns (FLH) and the cephalothoracic dorsolateral processes (CLDP). This assumption requires a detailed elaboration, which is limited here to the CLDP as an example, but can also be applied to the FLH: despite occasional attempts to homologise the cuticular processes in the Ancorabolidae as well as the *Ceratonotus* group (e.g., Conroy-Dalton and Huys 2000; Conroy-Dalton 2001; George 2020), no satisfactory analysis is available to date. With the standardised naming of the appendages, however, Lee and Huys (2019) have created a very important prerequisite for attempts of homology, which was further refined by George (2021). However, George’s (2020) statement that homology of the appendages requires comprehensive comparative studies still applies. Nevertheless, these could not be carried out within the scope of the present work but require their own detailed analysis. Yet, because George (2006a) postulated the lack of spinules on the cephalothoracic laterodorsal processes (CLDP) and the dorsal processes 1–4 (DP1–DP4) as an autapomorphy of *Pseudechinopsyllus*, an attempt was made here to subject the appendages in question to an intergeneric comparison. CLDP and DP, which are structurally almost identical and bear a sensillum at their tip, may be divided into five groups according to their size, shape and armouring: (i) small, conical, more or less rigid and naked (Fig. 15A), (ii) medium-sized, conical, more or less rigid and naked (Fig. 15B), (iii) medium- to large-sized, conical, more or less rigid and covered with many small spinules (Fig. 15C), (iv) large, conical, flexible and knobbed (Fig. 15D, (v) large, flexible, knobbed and dendroid (Fig. 15E). However, despite the similarity of the CLDP and DP, it was necessary to at least partially break up what in fact was a character complex, which is due to the fact that the CLDP are missing in *Pseudechinopsyllusandrei* sp. nov. We have therefore treated the CLDP and the DP1–DP4 complex separately.

CLDP: these processes are missing in *Dimorphipodia*, *Paratouphapleura*, *Polyascophorus*, *Poropsyllus*, and *Touphapleura* (cf. George 2021: fig. 15). The question of whether the CLDP developed as the last pair of dorsal processes – which would be suggested by the fact that ancestral representatives such as *Paratouphapleura* and *Touphapleura* lack them –, whether they were reduced secondarily – which could be the case in *Dimorphipodia*, *Polyascophorus* and *Poropsyllus* –, or whether both developmental pathways can be found in the *Ceratonotus* group, cannot be answered at this point. The spinule-less CLDP in *Pseudechinopsyllussindemarkae*, which were hypothesised here as homologous to those of the other CLDP-bearing taxa, correspond to the above-listed group (ii) and were considered a plesiomorphic state (compared to groups iii–v), whereas their absence in *P.andrei* sp. nov. was hypothesised as a secondary reduction (Table 2, character 14). *Pseudechinopsyllus* may be regarded as a rather derived taxon in the *Ceratonotus* group, which seems to be evident from the autapomorphies of the genus (Table 2, characters 1–6). *P.sindemarkae* shares the possession of CLDP with several other derived representatives of the *Ceratonotus* group, which, underpinned by their identical position on the cphth, suggests that these processes represent homologous structures, which were certainly inherited as synapomorphies from a last ancestor common only to their bearers. In contrast, there are presumably older taxa (*Touphapleura*, *Paratouphapleura*) in which the absence of the CLDP is probably a primary condition. This could be inferred from the fact that these more primitive taxa also exhibit a comparably primitive state with regard to other characteristics (e.g., the formation and size of DP1–DP3). If *Pseudechinopsyllus* were also assigned to these primitive representatives, then the lack of CLDP in *P.andrei* sp. nov. would have to be interpreted as primary, like in *Touphapleura* and *Paratouphapleura*. However, this would then mean that *P.sindemarkae* must have developed the processes convergent to the other CLDP-bearing taxa; its CLDP would not be homologous, but analogous to those of the other representatives and convergent as well as merely coincidentally developed at exactly the same attachment point on the cphth. This assumption, the primary absence of the CLDP in *P.andrei* sp. nov., and their convergent formation in *P.sindemarkae* seemed less plausible to us than their secondary regression in *P.andrei* sp. nov.

The same reasoning also applies to the FLH (character 13). Regarding the latter, however, it remains unclear at present how its absence in *Dorsiceratus*, a relatively derived genus, is to be interpreted.

In the present analysis, we assumed that a secondary reduction of the CLDP and FLH in *P.andrei* sp. nov. offers the most parsimonious explanation. Thus, characters 13 and 14 are also to be considered as autapomorphies of the new species.

Basing on the discussed autapomorphic characters 13–19, *P.andrei* sp. nov. can be justified as a distinct species.

### ﻿Examination of a closer relationship between *Pseudechinopsyllus* and *Echinopsyllus*

The phylogenetic analysis conducted by Wandeness et al. (2009), in which a sister-group relationship between *Echinopsyllus* and *Pseudechinopsyllus* was postulated, is not particularly robust in the opinion of Lee and Huys (2019). They based their doubts essentially on the assumption of Wandeness et al. (2009) that the anterior and posterior lateral processes on the Cphth (LII and LIII in Wandeness et al. (2009), CLVPa and CLVPp in Lee and Huys (2019) and here) should be interpreted as synapomorphies for *Echinopsyllus* and *Pseudechinopsyllus*. Lee and Huys (2019) correctly argued, however, that these processes are also formed in other representatives of the *Ceratonotus* group (indeed in *Ceratonotus*, *Dendropsyllus*, *Tauroceratus*). Though, Wandeness et al. (2009) had already admitted this and made their assumption subject to further studies. If, like Lee and Huys (2019), limiting oneself to the presumed homologous position of the processes on the cphth, one must agree with them, as Wandeness et al. (2009) had also only cited the presence/absence of the CLVPa respectively CLVPp as character states. We therefore took a closer look at the structure of the processes (Fig. 15A–E) and were able to include them in the analysis by modifying the character description of Wandeness et al. (2009) accordingly.

In comparison with the remaining representatives of the *Ceratonotus* group, we were able to identify five further potential synapomorphies of both genera in addition to six out of nine characters proposed by Wandeness et al. (2009) (Table 2, characters 31–41). Whether they will continue to be considered synapomorphies of *Echinopsyllus* and *Pseudechinopsyllus* in the future or must be assigned to a larger group of taxa of the *Ceratonotus* group will only be known after a comprehensive comparison of all representatives of the *Ceratonotus* group.

#### ﻿Character 31, Female body length > 900 µm:

A comparison of (as far as possible, mean) female body lengths (rostral tip to posterior end of FR) shows that *Echinopsyllus* and especially *Pseudechinopsyllus* are real giants within the *Ceratonotus* group (Table 3).

**Table 3.** (Mean) female body lengths (from rostral tip to furcal end) of the genera of the *Ceratonotus* group. The right-hand column shows the length difference to the 900 µm threshold.

Taxon	(Mean) body length (µm)	Difference to 900 µm
***Pseudechinopsyllus***	1,087	187
***Echinopsyllus***	989	89
***Arthuricornua***	744	-156
***Dimorphipodia***	641	-259
***Dorsiceratus***	630	-270
***Ceratonotus***	619	-281
***Dendropsyllus***	563	-337
***Poropsyllus***	550	-350
***Polyascophorus***	530	-370
***Paratouphapleura***	523	-377
***Tauroceratus***	520	-380
***Touphapleura***	520	-380
***Pseudopolyascophorus***	470	-430

Both genera clearly exceed the 900 µm mark (*Pseudechinopsyllus* even tops 1 mm), while the third largest size (Table 3, *Arthuricornua*) with 744 µm is more than 150 µm, the smallest (*Pseudopolyascophorus*) even 430 µm below the 900 µm mark. The representatives of the sister group of the *Ceratonotus* group, *Cletodes*, also have body lengths between 500 µm and 790 µm (e.g., Soyer 1964; Lang 1965; Dinet 1974; Por 1979; Schriever 1984; Wells and Rao 1987; Fiers 1991; George and Müller 2013) and thus do not reach the body size of *Echinopsyllus* and *Pseudechinopsyllus*.

In our opinion, the fact that *Echinopsyllus* and *Pseudechinopsyllus* of all species have such significantly larger body lengths is due to their common ancestry. The comparatively strong growth in size is therefore assumed to be a synapomorphy of both genera.

#### ﻿Characters 32, 34, CLVPa and CLVPp long, rigid, sclerotised:

The formation of anterior cephalothoracic lateroventral extensions (CLVEa) in the representatives of the *Ceratonotus* group was already recognised as essential by Conroy-Dalton (2001) and later treated in more detail by Lee and Huys (2019) and George (2021). In various taxa of the *Ceratonotus* group, they develop into more or less elongated CLVPa. Lee and Huys (2019) provided a description of the shape and size of the CLVPa and, as mentioned above, refuted the assumption of Wandeness et al. (2009) that the existence of these processes is a synapomorphy of *Echinopsyllus* and *Pseudechinopsyllus*. However, a comparison shows that both genera nevertheless differ from the other representatives with regard to the structure of the CLVPa (character 32). Only in *Echinopsyllus* and *Pseudechinopsyllus* are these processes – and the same applies equally to CLVPp (character 34) – developed as long, rigid, strongly sclerotised processes. In the other CLVPa- and CLVPp-carriers, however, they are completely different; they can be short and conical, long and rather tapered, or knobbed, or branched. Compared to *Echinopsyllus* and *Pseudechinopsyllus*, however, they always have a rather soft and apparently flexible structure. Their sclerotisation and the accompanying rigidity in the latter two genera is regarded here as a new formation. Following George (2021) we hypothesise that the CLVEa and CLVEp developed in different lineages into differently constructed CLVPa/CLVPp, which are assigned here to four diagnostic groups: (1) CLVEa but no CLVEP developed (*Arthuricornua*, *Dorsiceratus*; cf. Conroy-Dalton 2001; George 2006a; George and Plum 2009; George et al. 2025), (2) CLVEa not evolved into CLVPa, but CLVPp present (*Paratouphapleura*; cf. George 2021), (3) CLVEa not evolved into CLVPa, but the CLVPp long and forked (*Polyascophorus*, *Pseudopolyascophorus*; cf. Smirnov 1946; George 1998; George et al. 2013), (4) CLVPa evolved only weakly and the CLVPp evolved differently (*Ceratonotus*, *Dendropsyllus*, *Tauroceratus*; cf. George 2006b; George and Schminke 1998; Conroy-Dalton 2003b; Lee and Huys 2019; George 2021), (5) long rigid and sclerotised processes (*Echinopsyllus*, *Pseudechinopsyllus*; cf. Conroy-Dalton 2003a; George 2006a; Wandeness et al. 2009; present contribution). Based on this premise, we postulate the two process types, as present in *Echinopsyllus* and *Pseudechinopsyllus*, as a synapomorphic state.

#### ﻿Character 33, CLVPa basal TP displaced (sub)apically:

Lee and Huys (2019, p. 348) recognised an “extreme development” of the CLVPa in *Pseudechinopsyllus*, in which the originally basal tube pore was shifted to the tip of the process. We can also confirm this observation for *P.andrei* sp. nov. (Fig. 4A). However, because they had excluded *Echinopsyllus* from their comparison, Lee and Huys (2019) missed the fact that a (sub)apical shift of the tube pore also took place in that genus. This is a further indication of the close relationship between *Echinopsyllus* and *Pseudechinopsyllus* and is interpreted by us as synapomorphic for both genera.

#### ﻿Character 35, DP4 developed:

The dorsal processes on the P5-bearing thoracic somite (DP4) are only developed in seven of the 13 genera of the *Ceratonotus* group (cf. Lee and Huys 2019; George 2021), including *Echinopsyllus* and *Pseudechinopsyllus*. However, although it can be assumed that this is at least a positional homology, it remains unclear whether the presence of the DP4 actually represents a synapomorphy of the seven genera, which otherwise differ very clearly from each other. Only a comprehensive phylogeny of the *Ceratonotus* group will be able to shed light on this. In the meantime, we postulate that the new formation of DP4 represents a further common derivation (synapomorphy) of *Echinopsyllus* and *Pseudechinopsyllus*.

#### ﻿Characters 36–39, deviations on the mouthparts:

Although the mouthparts offer a number of comparable characters, they are not often considered in phylogenetic studies. This is mainly due to the fact that because they are small and difficult to prepare, they are sometimes described only inaccurately, incompletely, or not at all in (especially older) species descriptions. For the present study, however, some characters could be included. The setae of the mandibular palpus, for example, provide promising clues. George (2021: fig. 19) attempted to discover possible indications of relationships in the number of setae on the md palpus. And this shows that *Echinopsyllus* and *Pseudechinopsyllus* are the only representatives of the *Ceratonotus* group to have completely reduced setae nos. 1 and 6. The loss of one of the two setae can be observed several times and points – apart from the possibility of an always independent and therefore convergent loss of the setae – to two possible lines of development (cf. George 2021, fig. 19). Thus, *Echinopsyllus* and *Pseudechinopsyllus* share the reduction of seta no. 1 (character 36) with *Dorsiceratus* and *Dimorphipodia* – which was interpreted as convergence by George et al. (2025) –, and that of seta no. 6 (character 37) with *Arthuricornua*, *Ceratonotus*, *Dendropsyllus*, *Polyascophorus*, *Poropsyllus*, *Pseudopolyascophorus* and *Tauroceratus*. However, regardless of which of the three possibilities proves to be the correct one, the loss of the two setae can be assumed to be a synapomorphy of the two genera. Until final clarification, we list both characters as synapomorphies, which nevertheless also occur at least convergently in other taxa of the *Ceratonotus* group.

*Echinopsyllus* and *Pseudechinopsyllus* are characterised by one apical seta on the coxa of the mxl (character 38). They share that derived state with one further species, *Pseudopolyascophorusmonoceratus*, whereas all remaining taxa of the *Ceratonotus* group still bear two setae. However, analogous to the here presumed convergent extension of the anterior rostral region (see above, character 7), because *Pse.monoceratus* does not share any of the other synapomorphies of the two genera, which in turn do not have any of the autapomorphies of *Pse.monoceratus*, the reduction of one of the two apical setae is regarded as convergent and assumed to be a synapomorphy of *Echinopsyllus* and *Pseudechinopsyllus*.

*Echinopsyllus* and *Pseudechinopsyllus* are the only representatives of the *Ceratonotus* group that have the seta on the maxillipedal syncoxa completely reduced (character 39). All other taxa bear a seta apically. This reduction is an undoubted synapomorphy of *Echinopsyllus* and *Pseudechinopsyllus*.

#### ﻿Character 40, a tube pore on the P2 exp2:

We have already mentioned the occurrence of tube pores several times, as well as the occasional difficulty in recognising patterns that could indicate relatedness. Tube pores are relatively common on the last segments (exp3) of P2 and P3, and less frequently on those of P1 and P4. *Echinopsyllus* and *Pseudechinopsyllus* show a peculiarity: in them, tube pores are detectable at the P2 exp2 (character 40). This is unique within the *Ceratonotus* group and an undoubted synapomorphy of both genera.

#### ﻿Character 41, formation of an extremely long setophore:

Although *Polyascophorus*, *Pseudopolyascophorus*, and *Touphapleura* also show a strong elongation of the setophore (George 1998; George et al. 2013), it never exceeds the length of the P5 exopod. This is nevertheless the case with all *Echinopsyllus* and *Pseudechinopsyllus* species: the setophore is significantly longer than, and in some cases almost twice as long as, the exopod. Like character 40, character 41 can therefore also be regarded as a clear synapomorphy of the two genera.

In summary, it can be stated that characters 31–41 provide sufficient and meaningful evidence for a sister group relationship between *Echinopsyllus* and *Pseudechinopsyllus*.

In addition to the few characters listed here as potential convergences, there are a number of other derived features that characterise both *Echinopsyllus* and *Pseudechinopsyllus*. However, they also occur, sometimes very scattered, in other representatives of the *Ceratonotus* group. Examples include the complete degeneration of the endopods of P1 and/or P2, which can be observed in different taxa (*Arthuricornua*, *Dimorphipodia*, *Echinopsyllus*, and *Pseudechinopsyllus* (P1 and P2 without endopod); *Dendropsyllus*, *Pseudopolyascophorus*, and *Tauroceratus* (part.) (P2 without endopod)). It is not clear at this stage whether this high-quality derivation can be attributed to the descent of all involved taxa from a last common ancestor, or whether a convergent regression of the endopod(s) is to be assumed here as well.

## ﻿Conclusions

The description of a new species of the taxon *Pseudechinopsyllus* George, 2006 (Copepoda, Harpacticoida, Cletodidae) served as the basis for a revision of the genus and the substantiation of its close relationship with *Echinopsyllus* Sars, 1909. *Pseudechinopsyllusandrei* sp. nov. from the Campos Basin (Brazil) could be established as a distinct species on the basis of seven autapomorphies. In addition, six synapomorphies justify its sister group relationship to *P.sindemarkae* George, 2006, which at the same time confirms the taxon *Pseudechinopsyllus* as a monophylum.

A comparison with the four so-far known species of the taxon *Echinopsyllus* as well as with the remaining representatives of the *Ceratonotus* group sensu Conroy-Dalton (2001), a monophylum inside the Cletodinae T. Scott, 1905, yielded eleven further synapomorphies that support the sister group relationship of the genera *Pseudechinopsyllus* and *Echinopsyllus*. The study contributes to the clarification of systematics within the *Ceratonotus* group.

## Supplementary Material

XML Treatment for Pseudechinopsyllus

XML Treatment for Pseudechinopsyllus andrei

## ﻿Additional information

### Author ORCIDs

Kai Horst George https://orcid.org/0000-0001-6464-0099

Adriane P. Wandeness https://orcid.org/0000-0003-3193-5784

Paulo J. P. Santos https://orcid.org/0000-0003-4826-362X

### Data availability

All of the data that support the findings of this study are available in the main text.
